# Brain fatty acid and transcriptome profiles of pig fed diets with different levels of soybean oil

**DOI:** 10.1186/s12864-023-09188-6

**Published:** 2023-02-28

**Authors:** Bruna Pereira Martins da Silva, Simara Larissa Fanalli, Julia Dezen Gomes, Vivian Vezzoni de Almeida, Heidge Fukumasu, Felipe André Oliveira Freitas, Gabriel Costa Monteiro Moreira, Bárbara Silva-Vignato, James Mark Reecy, James Eugene Koltes, Dawn Koltes, Júlio Cesar de Carvalho Balieiro, Severino Matias de Alencar, Julia Pereira Martins da Silva, Luiz Lehmann Coutinho, Juliana Afonso, Luciana Correia de Almeida Regitano, Gerson Barreto Mourão, Albino Luchiari Filho, Aline Silva Mello Cesar

**Affiliations:** 1 Faculty of Animal Science and Food Engineering, University of São Paulo, Pirassununga, São Paulo, Brazil; 2 Luiz de Queiroz College of Agriculture, University of São Paulo, Piracicaba, São Paulo, Brazil; 3 College of Veterinary Medicine and Animal Science, Federal University of Goiás, Goiânia, Goiás Brazil; 4 Unit of Animal Genomics, University of Liège, GIGA Medical Genomics, Liège, Belgium; 5 College of Agriculture and Life Sciences, Iowa State University, Ames, IA USA; 6 School of Veterinary Medicine and Animal Science, University of São Paulo, Pirassununga, São Paulo, Brazil; 7 Embrapa Pecuária Sudeste, São Carlos, São Paulo, Brazil

**Keywords:** Immune response, Soybean oil, Calcium transport, Lipid metabolism, Oxidative processes, Pigs

## Abstract

**Background:**

The high similarity in anatomical and neurophysiological processes between pigs and humans make pigs an excellent model for metabolic diseases and neurological disorders. Lipids are essential for brain structure and function, and the polyunsaturated fatty acids (PUFA) have anti-inflammatory and positive effects against cognitive dysfunction in neurodegenerative diseases. Nutrigenomics studies involving pigs and fatty acids (FA) may help us in better understanding important biological processes. In this study, the main goal was to evaluate the effect of different levels of dietary soybean oil on the lipid profile and transcriptome in pigs’ brain tissue.

**Results:**

Thirty-six male Large White pigs were used in a 98-day study using two experimental diets corn-soybean meal diet containing 1.5% soybean oil (SOY1.5) and corn-soybean meal diet containing 3.0% soybean oil (SOY3.0). No differences were found for the brain total lipid content and FA profile between the different levels of soybean oil. For differential expression analysis, using the DESeq2 statistical package, a total of 34 differentially expressed genes (DEG, FDR-corrected p-value < 0.05) were identified. Of these 34 DEG, 25 are known-genes, of which 11 were up-regulated (log2 fold change ranging from + 0.25 to + 2.93) and 14 were down-regulated (log2 fold change ranging from − 3.43 to -0.36) for the SOY1.5 group compared to SOY3.0. For the functional enrichment analysis performed using MetaCore with the 34 DEG, four pathway maps were identified (*p*-value < 0.05), related to the *ALOX15B* (log2 fold change − 1.489), *CALB1* (log2 fold change − 3.431) and *CAST* (log2 fold change + 0.421) genes. A “calcium transport” network (*p*-value = 2.303e-2), related to the *CAST* and *CALB1* genes, was also identified.

**Conclusion:**

The results found in this study contribute to understanding the pathways and networks associated with processes involved in intracellular calcium, lipid metabolism, and oxidative processes in the brain tissue. Moreover, these results may help a better comprehension of the modulating effects of soybean oil and its FA composition on processes and diseases affecting the brain tissue.

**Supplementary Information:**

The online version contains supplementary material available at 10.1186/s12864-023-09188-6.

## Background

The pigs (*Sus scrofa*) have global economic impact as it is the second most consumed meat-based protein source worldwide [1, 2]. Additionally, pigs are considered an animal model and have been used in research in the area of nutrigenomics and human metabolic diseases. Moreover, pigs can be used to understand neurodegenerative diseases due to similar of the brain anatomy, development, function, and neurophysiological process compared to the brains of small laboratory animals and humans [3–6].

The brain contains high lipid content, making up approximately 50% of the brain’s dry weight, only lower than the adipose tissue [7]. Lipids are essential for brain structure and function, and the central nervous system is fundamental for the regulation of metabolism and lipid balance [8, 9]. In addition, some regions of the brain are capable to detect nutrients and hormones that regulate energy balance and feeding [8, 9].

A noteworthy factor is that the diet fed to the pigs can alter the lipid and fatty acids (FA) profiles of the tissues [10, 11]. Thus, soybean oil has been commonly used as part of the feed composition for growing-finishing pigs because it results in improved growth performance and beneficial effects to consumers [12]. In addition, soybean oil is high in polyunsaturated fatty acids (PUFA), being rich in linolenic acid (LA, C18:2 n-6), which is associated with the reduction of cardiovascular diseases and serum cholesterol [13].

Dietary derived FA, such as LA and alpha-linolenic acid (ALA, C18:3 n-3), act as precursors of PUFA like docosahexaenoic acid (DHA, C22:6 n-3) and arachidonic acid (AA, C20:4 n-6). Dietary supplementation of DHA may have potential neuroprotection effects against chronic and acute inflammation in the central nervous system, as well as slowing cognitive decline in Alzheimer’s disease [14]. PUFA and their metabolites act in the brain by activating receptors and cell signaling pathways. Additionally, they are responsible for modulating the system related to signaling lipids, present in phospholipids of the neuronal cell membrane, and for regulating synaptic function [15, 16].

While the roles of specific classes of FA in brain function are being elucidated, the understanding of the genes involved in the dietary modulation of FA in the brain is unclear and limited. Thus, the objective of this work was to determine if different levels of dietary soybean oil fed to male pigs would modify the lipid and transcriptome profile of the brain.

## Results

### Total lipid content and FA profile

Table 1 shows the total lipid composition and FA profile of the brain tissue from pigs given diets with different levels of soybean oil (SOY1.5 vs. SOY3.0). No changes (*p*-value ≤ 0.05) were identified in the total lipid content and the FA profile between the treatments.

**Table 1:** Total lipid content and FA profile in brain tissue of pigs fed diets containing different levels of soybean oil

Fatty acid, %	Dietary treatment^1^		Pooled SEM^2^	*p-*value
	SOY1.5	SOY3.0		
Total lipids	9.928	10.292	0.113	0.199
Saturated fatty acid (SFA)				
Myristic acid (C14:0)	0.522	0.521	0.006	0.927
Palmitic acid (C16:0)	26.848	27.037	0.189	0.709
Stearic acid (C18:0)	29.131	28.371	0.208	0.110
Monounsaturated fatty acid (MUFA)				
Palmitoleic acid (C16:1)	0.494	0.462	0.015	0.387
Oleic acid (C18:1 n-9)	30.071	29.955	0.143	0.678
Eicosenoic acid (C20:1 n-9)	1.897	1.898	0.024	0.967
Polyunsaturated fatty acid (PUFA)				
Linoleic acid (C18:2 n-6)	2.321	2.309	0.262	0.984
Alpha-linolenic acid (C18:3 n-3)	ND^3^	ND	-	-
Eicosapentaenoic acid (C20:5 n-3, EPA)	0.141	0.135	0.006	0.759
Docosahexaenoic acid (C22:6 n-3, DHA)	8.781	8.926	0.151	0.620
Total SFA	56.584	55.925	0.277	0.396
Total MUFA	32.494	32.501	0.192	0.987
Total PUFA	10.852	11.685	0.240	0.062
Total n-3 PUFA^4^	8.705	9.014	0.125	0.136
Total n-6 PUFA^5^	1.806	1.768	0.097	0.901
PUFA:SFA ratio^6^	0.192	0.207	0.005	0.134
n-6:n-3 PUFA ratio^7^	0.210	0.231	0.018	0.607
Atherogenic index^8^	0.664	0.661	0.008	0.921

^1^Pigs (*n* = 36; 18 pigs/treatment) were fed either a corn-soybean meal diet containing 1.5% soybean oil (SOY1.5) or diets containing with 3.0% soybean oil (SOY3.0). Values represent the least square means. ^2^SEM = standard error of the least square means. ^3^ND = not detected. ^4^Total n-3 PUFA = {[C18:3 n-3] + [C20:5 n-3] + [C22:6 n-3]}. ^5^Total n-6 PUFA = C18:2 n-6. ^6^PUFA:SFA ratio = total PUFA/total SFA. ^7^Σ n-6/Σ n-3 PUFA ratio. ^8^Atherogenic index = (4 × [C14:0]) + (C16:0)/(total MUFA] + [total PUFA]), where brackets indicate concentrations [17].

### RNA-Seq data and differentially expressed genes

An average number of total reads per sample of 33.4 M and 32.9 M, was obtained for the SOY1.5 group, before and after quality control, respectively. For the SOY3.0 group, the average number of sequenced reads, before and after quality control, were 34.3 M and 33.9 M, respectively. Of the total reads obtained for both groups, after quality control, 95.02% of them reads were mapped against the reference genome *SScrofa11.1* (Additional file 1, Table S1).

Differential analysis was performed comparing the level of gene expression between the groups, and a total of 22,931 genes were identified in the brain tissue. Of this 34 were DEG (FDR-corrected *p*-value < 0.05). Within the 34 DEG, 25 were known-genes, 11 being up-regulated (log2 fold change ranging from + 0.25 to + 2.93) and 14 being down-regulated (log2 fold change ranging from − 3.43 to -0.36) in the SOY1.5 compared to the SOY3.0. The genes with the most altered expression were *CALB1* (log2 fold change − 3.43; FDR = 0.03) and *VMO1* (log2 fold change + 2.93; FDR < 0.01). The list of expressed genes and DEG are demonstrated in Table S2.

### Functional enrichment analysis

The MetaCore software was used to identify pathway maps from the list of 34 DEG (FDR < 0.05). Four pathway maps were identified (*p*-value < 0.05), related to the following genes: arachidonate 15-lipoxygenase type B (*ALOX15B*), calbidin-1 (*CALB1*), and calpastatin (*CAST*), as shown in Table 2.

**Table 2:** Pathway maps SOY1.5^1^ vs. SOY3.0^2^ in brain tissue of pigs fed diets containing different levels of soybean oil

Pathway map	*p*-value	DEG^3^	log2 fold change
Linoleic acid metabolism	1.970e-02	*ALOX15B*	-1.489
Prostaglandin-1 biosynthesis and metabolism	3.597e-02	*ALOX15B*	-1.489
Renal secretion of inorganic electrolytes	3.721e-02	*CALB1*	-3.431
Immune response_IL-5 signaling via PI3K, MAPK, and NF-kB	4.77e-02	*CAST*	+ 0.421

^1^SOY1.5: corn-soybean meal diet containing 1.5% soybean oil. ^2^SOY3.0: corn-soybean meal diet containing 3.0% soybean oil. ^3^DEG: Differentially expressed genes.

**Fig. 1:**
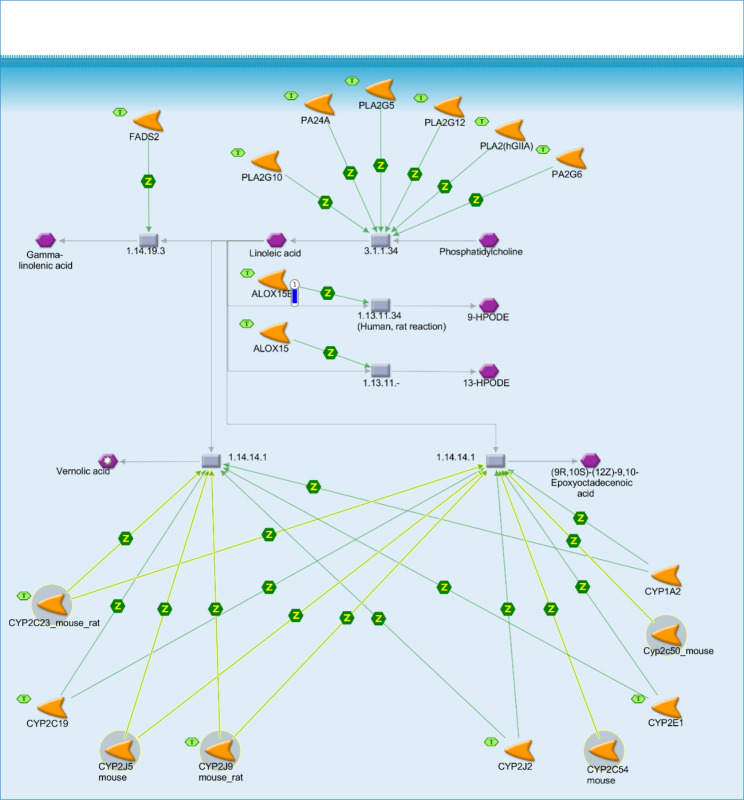
Linoleic acid metabolism in brain tissue of pigs fed diets containing different levels of soybean oil (SOY1.5^1^ vs. SOY3.0^2^). ^1^SOY1.5: corn-soybean meal diet containing 1.5% soybean oil. ^2^SOY1.5: corn-soybean meal diet containing 3.0% soybean oil. The experimental data is represented by the thermometer-like figure on the map. The downward thermometer (blue) indicates down-regulation of the *ALOX15B* DEG (log2 fold change − 1.489) in the SOY1.5 group compared to SOY3.0. Network objects are represented by individual symbols. The green “T” icon shows which object is associated with the brain tissue. Interactions between objects are represented by arrows, mechanisms, and logical relationships. Further explanations are provided at https://portal.genego.com/legends/MetaCoreQuickReferenceGuide.pdf.

**Fig. 2:**
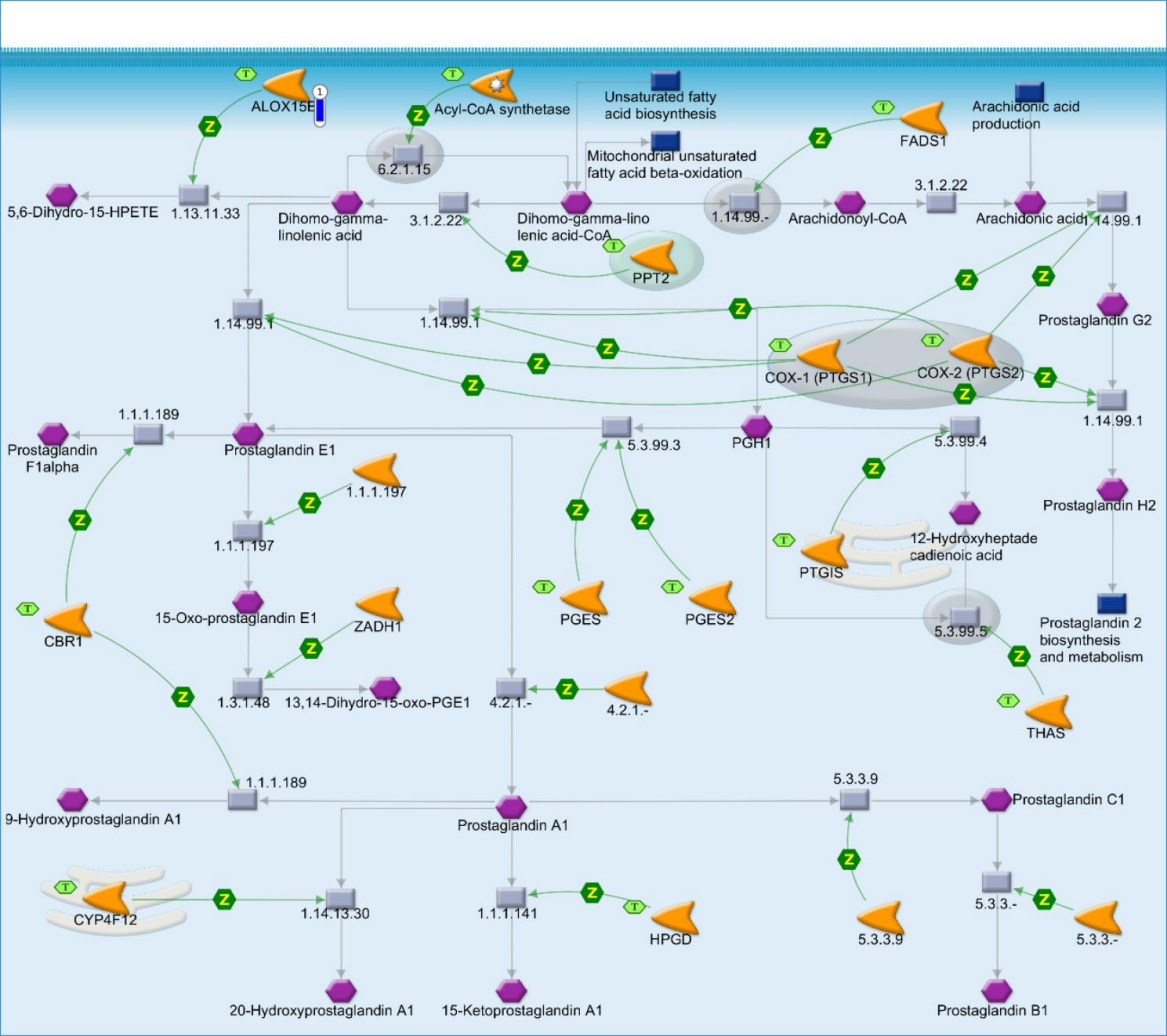
Prostaglandin-1 biosynthesis and metabolism in brain tissue of pigs fed diets containing different levels of soybean oil (SOY1.5^1^ vs. SOY3.0^2^). ^1^SOY1.5: corn-soybean meal diet containing 1.5% soybean oil. ^2^SOY1.5: corn-soybean meal diet containing 3.0% soybean oil. The experimental data is represented by the thermometer-like figure on the map. The downward thermometer (blue) indicates down-regulation of the *ALOX15B* DEG (log2 fold change − 1.489) in the SOY1.5 group compared to SOY3.0. Network objects are represented by individual symbols. The green “T” icon shows which object is associated with the brain tissue. Interactions between objects are represented by arrows, mechanisms, and logical relationships. Further explanations are provided at https://portal.genego.com/legends/MetaCoreQuickReferenceGuide.pdf.

The *ALOX15B* DEG, showing a down-regulation in the SOY1.5 group compared to SOY3.0 (log2 fold change − 1.489). The *ALOX15B*, participate in two of the four significant enriched pathway maps identified: “Linoleic acid metabolism” (*p*-value = 1.970e-2, Fig. 1), and “Prostaglandin-1 biosynthesis and metabolism” (*p*-value = 3.597e-2, Fig. 2).

**Fig. 3:**
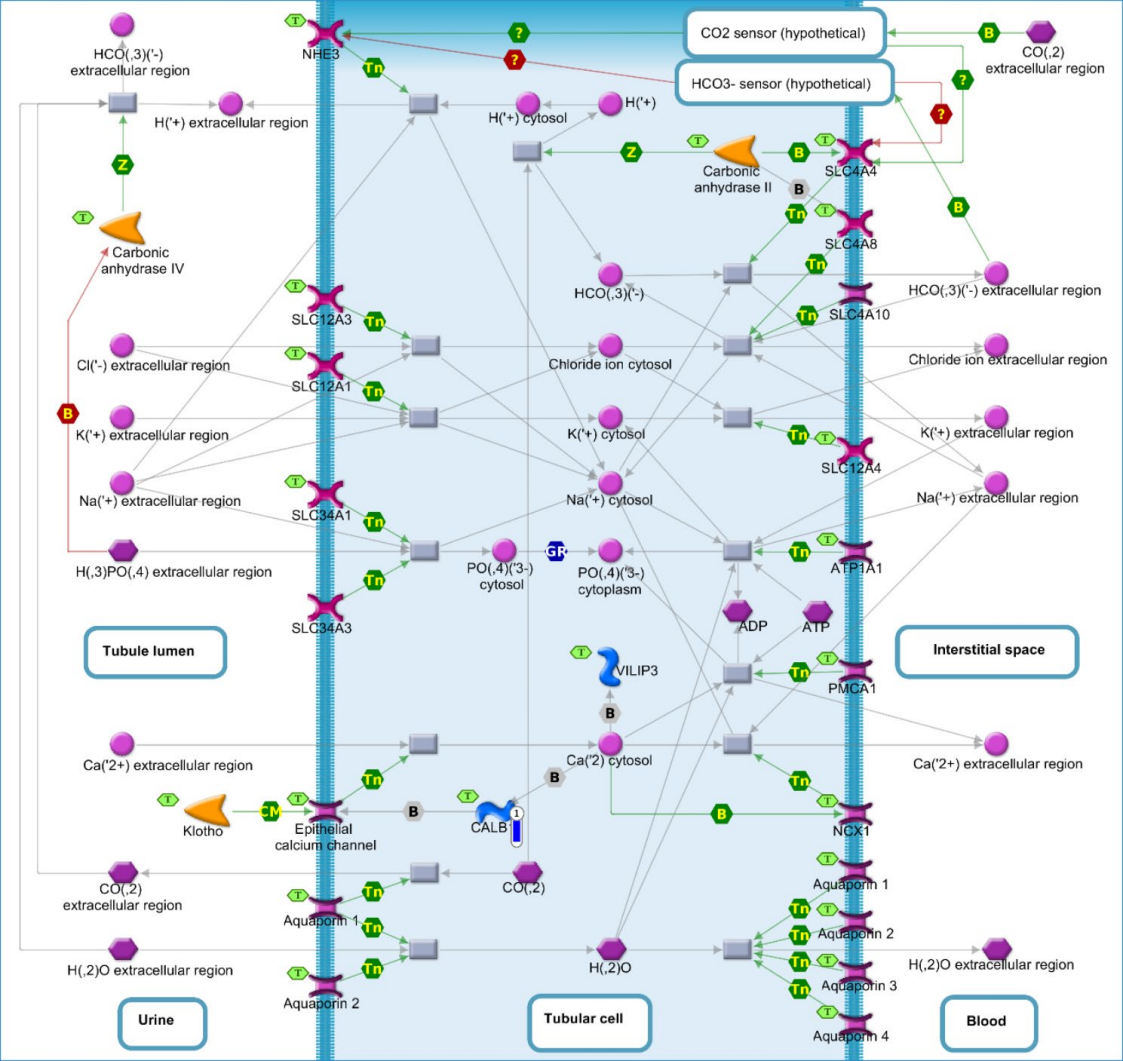
Renal secretion of inorganic electrolytes in brain tissue of pigs fed diets containing different levels of soybean oil (SOY1.5^1^ vs. SOY3.0^2^). ^1^SOY1.5: corn-soybean meal diet containing 1.5% soybean oil. ^2^SOY1.5: corn-soybean meal diet containing 3.0% soybean oil. The experimental data is represented by the thermometer-like figure on the map. The downward thermometer (blue) indicates down-regulation of the *CALB1* DEG (log2 fold change − 3.431) in the SOY1.5 group compared to SOY3.0. Network objects are represented by individual symbols. The green “T” icon shows which object is associated with the brain tissue. Interactions between objects are represented by arrows, mechanisms, and logical relationships. Further explanations are provided at https://portal.genego.com/legends/MetaCoreQuickReferenceGuide.pdf.

The *CALB1* DEG, showing a down-regulation in the SOY1.5 group compared to SOY3.0 (log2 fold change − 3.431). The *CALB1*, participates in the enriched pathway map “Renal secretion of inorganic electrolytes” (*p*-value = 3.721e-2, Fig. 3).

**Fig. 4:**
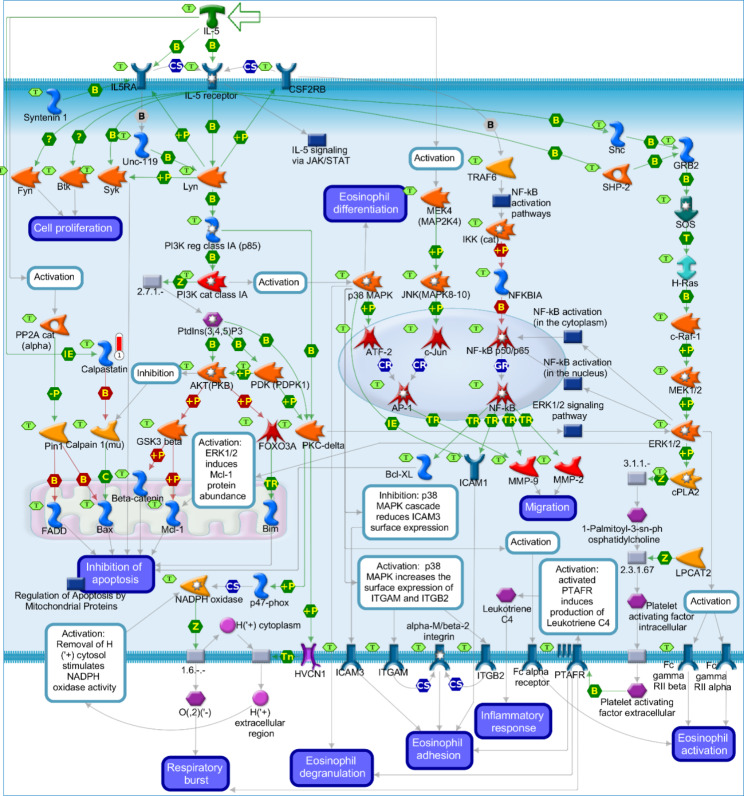
Immune response_IL-5_signaling in brain tissue of pigs fed diets containing different levels of soybean oil (SOY1.5^1^ vs. SOY3.0^2^). ^1^SOY1.5: corn-soybean meal diet containing 1.5% soybean oil. ^2^SOY1.5: corn-soybean meal diet containing 3.0% soybean oil. The experimental data is represented by the thermometer-like figure on the map. The upward thermometer (red) indicates up-regulation of the *CAST* DEG (log2 fold change + 0.421) in the SOY1.5 group compared to SOY3.0. Network objects are represented by individual symbols. The green “T” icon shows which object is associated with the brain tissue. Interactions between objects are represented by arrows, mechanisms, and logical relationships. Further explanations are provided at https://portal.genego.com/legends/MetaCoreQuickReferenceGuide.pdf.

The *CAST* DEG, showing an up-regulation in the SOY1.5 group compared to SOY3.0 (log2 fold change + 0.421). The *CAST* participates in the enriched pathway map “Immune response IL-5 signaling via PI3K, MAPK, and NF-kB” (*p*-value = 4.770e-2, Fig. 4).

**Fig. 5:**
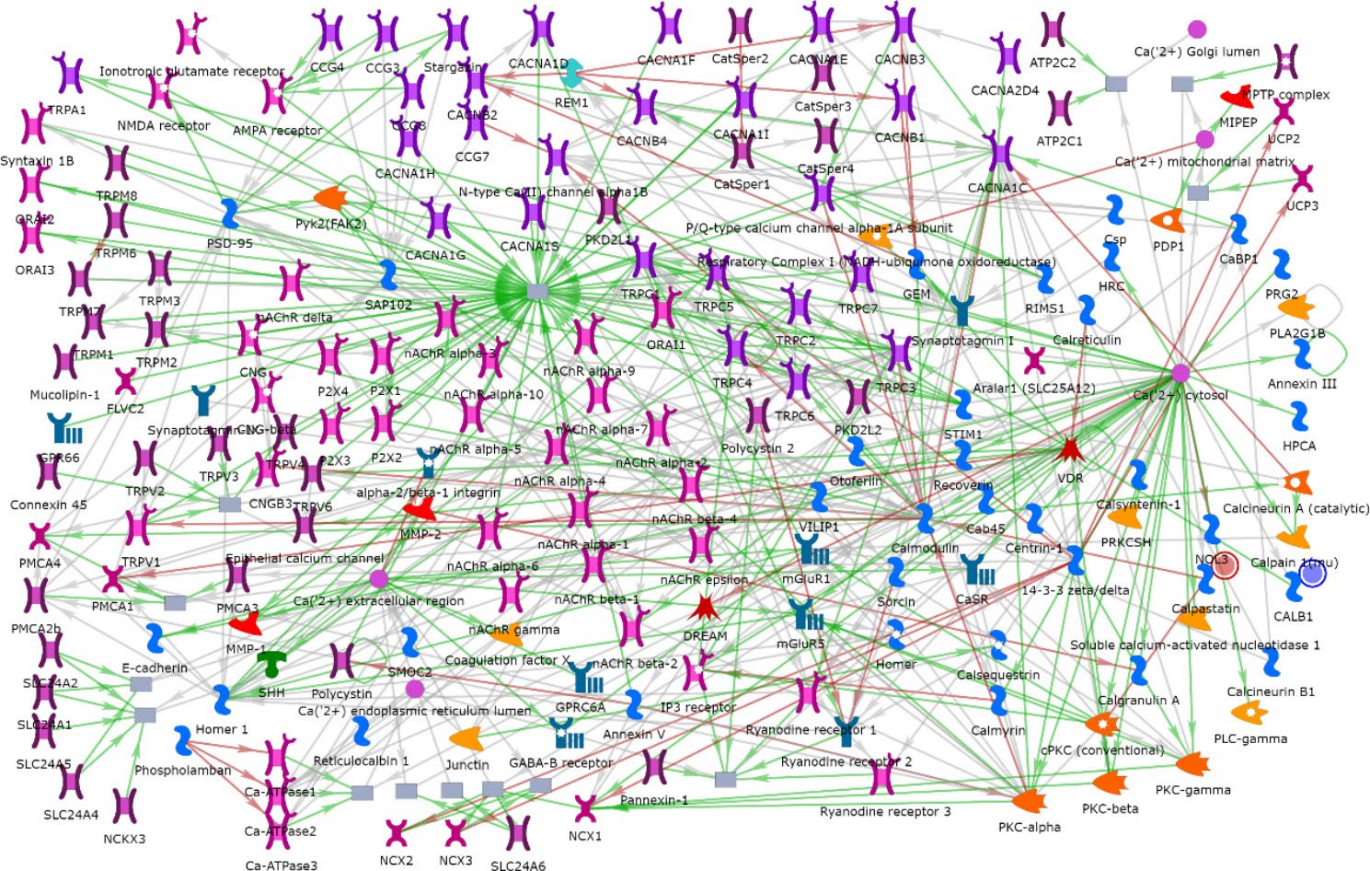
Calcium transport in brain tissue of pigs fed diets containing different levels of soybean oil (SOY1.5^1^ vs. SOY3.0^2^). ^1^SOY1.5: corn-soybean meal diet containing 1.5% soybean oil. ^2^SOY1.5: corn-soybean meal diet containing 3.0% soybean oil. The experimental data are represented by the intensity of the blue and red circles on the network. The blue circle indicates down-regulation of the *CALB1* DEG, and the red circle indicates up-regulation of the *CAST* DEG SOY1.5 group compared to SOY3.0. Green arrows indicate positive interactions, red arrows indicate negative interactions, and gray arrows indicate unspecified interactions. Further explanations are provided at https://portal.genego.com/legends/MetaCoreQuickReferenceGuide.pdf.

To better understand the behavior of the genes and their interactions, process networks were additionally generated by using the MetaCore software. The “Calcium transport” process network (*p*-value = 2.303e-2), was the only network detected herein, containing the DEG *CALB1* (log2 fold change − 3.431) and *CAST* (log2 fold change + 0.421) (Fig. 5).

## Discussion

No changes were identified in the total lipid content and the FA profile between the treatments. The results found in the functional enrichment analysis, demonstrated that the use of different levels of soybean oil alters the transcriptomic profile of pig brain, affecting key processes for the well-functioning of this tissue. For the enriched pathways illustrated in Figs. 1 and 2, the *ALOX15B* participates in lipid oxidation and peroxidation reactions. According to Stelzer et al. [18], among the pathways associated with this gene there were “eicosanoid synthesis” and “arachidonic acid metabolism” and the related Gene Ontology (GO) annotations include “calcium ion binding” and “lipid binding”. Fanalli et al. [19] demonstrated that the addition of soybean oil in the diet of pigs in different proportions acted on the modulation of genes, pathway maps and networks associated with inflammation, immune response, oxidative stress, and neurodegenerative diseases, in muscle and liver.

Lipoxygenases (LOX) are a family of enzymes responsible for the oxidation of lipids and the generation of a range of metabolites such as eicosanoids and PUFA-related compounds. These metabolites play diverse physiological and pathological roles in inflammatory, neurodegenerative, and cardiovascular diseases, as well as, in defence mechanisms [20, 21]. Lipoxygenases have also been reported in cell differentiation [22, 23], apoptosis [24], and play an important role in the immune response by helping to regulate cytokine secretion [25].

Among the LOX reported in mammals, the *ALOX15* isoform may oxygenate complex lipid-protein assemblies found in biomembranes and lipoproteins [26]. The *ALOX15* also binds to membranes, with intracellular calcium as a main cofactor for this interaction [27, 28]. It has been reported that *ALOX15* is expressed at higher levels in human airway epithelial cells, in eosinophils and immature red blood cells [29]. Furthermore, according to van Leyen et al. [30] and Han et al. [31], expression and regulation of *ALOX15* transcription also occurs in various areas of the brain, but at lower levels. In the study of Shalini et al. [32], a higher expression of *ALOX15* mRNA was found in the prefrontal cortex.

The main product of AA oxygenation by *ALOX15/15B* is 15-hydroxyeicosatetraenoic (*15-HETE*) [33]. The *15-HETE* is considered an important precursor of specialized pro-resolving lipid mediators and is associated with pro- and anti-inflammatory effects [34, 35]. It has also been reported that *15-HETE* is a ligand and activator of the peroxisome proliferator-activated receptor gamma (*PPAR-γ*), which at high concentrations may generate reactive oxygen species in cells [36, 37], and may induce the production of the pro-inflammatory cytokine Interleukin-12 (IL-12) [35, 38]. Zhan et al. [39] demonstrated that the application of flaxseed-enriched diet (rich in n-3 PUFA, similar to soybean oil), showed decreases in the expression of pro-inflammatory cytokine genes through activation of *PPAR-γ* in muscle, adipose tissue and spleen of growing-finishing barrows.

Among the results of DHA oxidation by *ALOX15*, are the specialized pro-resolving lipid mediator D5, a mediator that may be associated in the resolution of inflammation and in the regulation of immune response [40]. Another important mediator related to the resolution of inflammation, reduction of leukocyte trafficking, and negative regulation of cytokine expression is neuroprotectin D1 (NPD1) [41, 42]. NPD1 is reported as an anti-inflammatory molecule, which acts in neuroplasticity and brain signaling, and when in altered conditions, may be found in neuroinflammatory disorders and chronic neurodegeneration [32].

In the study of Chaung et al. [43] dietary supplementation of phosphatidylserine and DHA improved antioxidant activity and cognitive function (spatial memory) in rat pups during brain development. Richter et al. [44] demonstrated that daily intake of soy-derived phosphatidylserine, had positive effects on cognitive function (learning and memory) in elderly people with impaired memory function.

The *ALOX15* was found to have increased expression in the brains of Alzheimer’s patients [26, 45, 46]. Praticò et al. [45], reported higher levels of *12/15-LOX* and its metabolites *12/15(S)-HETE* in the temporal and frontal brain regions of Alzheimer’s patients. It was further found in in vitro studies using neuronal cells with Alzheimer’s mutation, that *12/15-LOX* is associated with regulation of tau phosphorylation and Aβ plaque production. In addition, regulates synaptic pathology associated with behavioral deficiencies [47, 48].

Additionally, studies have shown that 12/15-LOX is crucial in Parkinson’s disease [49–51]. According to the research of Li et al. [49] and Canals et al. [50], activation of these isoforms was associated with a decrease in glutathione concentration (a marker of Parkinson’s disease) in neurons, which may induce nitric oxide neurotoxicity and damage to dopaminergic neurons. The mechanism of action of 12/15-LOX is still unclear. For example, inhibiting 12/15-LOX has been shown to reduce reactive oxygen species-induced neuronal cell death [51]. Other studies found that 12/15-LOX and its metabolites have both pro- and anti-inflammatory effects. This controversial nature of 12/15-LOX has been reported to be dependent on the metabolites produced, the site of inflammation, and the levels of these metabolites produced [35]. The brain is a tissue that contains a wide range of metabolites and in distinct concentrations. Thus, due to the controversial nature of *ALOX15B* in metabolic and oxidative processes, further investigations are needed to understand the influence of the downregulation of this DEG in the SOY1.5 group that was found in our study. Further research is required to confirm the action of *ALOX15B* in the progression of neurodegenerative and inflammatory diseases.

For the enriched pathway in Fig. 3, the *CALB1* gene binds to intracellular calcium transported via the epithelial calcium channel and transports it across the cytosol toward the basolateral membrane [52]. As a protein-encoding gene that participates in calcium transport, GO annotations for the *CALB1* are found to be related to “calcium ion binding” and “vitamin D binding” [18]. It is a highly conserved calcium-binding protein that belongs to a family of high-affinity calcium-binding proteins [53, 54]. Furthermore, studies have shown that the *CALB1* gene is highly expressed in brain tissue and found in the majority of neuronal cells and that it is not vitamin D dependent [53–55].

Calcium is one of the most important signaling factors and acts to regulate several important cellular functions such as growth, differentiation, proliferation, cell survival and apoptosis, membrane excitability, and gene transcription. Calcium is also essential for maintaining normal brain function [56]. Thus, the dysregulation of calcium homeostasis and endoplasmic reticulum stress is associated with several pathological conditions such as Parkinson’s, Huntington’s, and Alzheimer’s diseases, and affects numerous signaling pathways [56, 57]. This pathogenic event may also cause amyloidogenesis, energy deficits in neurons, protein aggregation and oxidative stress, and changes in mitochondrial dysfunction, plasticity, and synaptic transmission [58].

Disturbed mitochondrial calcium regulation may also be associated with the link between neuronal dysfunction and disruption of the mitochondria-associated membrane (MAM) contact site of the endoplasmic reticulum and mitochondria, since calcium acts to modulate neurotransmitter release during the synapse [59]. This dysregulation of the MAM-mitochondria linkage dysfunction may also be associated with neurodegenerative diseases such as Alzheimer’s disease [59]. The MAMs are regions of the endoplasmic reticulum that mediate communication between the reticulum and the mitochondria [59, 60]. They are regions that are involved in calcium transport, are responsible for several lipid biosynthetic enzymatic activities, and are also a strategic site for lipid metabolism [59, 61, 62]. According to Vance [59], defects associated with these regions have been identified in neurodegenerative diseases and insulin resistance/type 2 diabetes.

The *CALB1* helps maintain calcium homeostasis, regulate intracellular calcium responses to physiological stimuli, and modulating synaptic transmission [54]. Another important role of *CALB1*, is its action in the prevention of neuronal death [54, 63]. The *CALB1* also plays an important role in buffering cytosolic calcium and helps prevent lipid peroxidation, through its expression in pancreatic-β cells, by eliminating the production of lipid hydroperoxide, which is induced by proinflammatory cytokines [64]. There is evidence that *CALB1* acts to protect neurons against calcium-mediated neurotoxicity and may be considered a cytochemical marker for neuronal plasticity [55].

Decreases in *CALB1* expression/concentration in brain tissue has been associated with neurodegeneration in Alzheimer’s, Parkinson’s, and Huntington’s diseases [18, 65] and in ischemic injury studies [66, 67]. Lower *CALB1* expression has also been associated with a higher rate of neuronal death [68]. Increased expression of *CALB1*, on the other hand, has been reported to induce neurite growth in dopaminergic neuronal cells, demonstrating its protective role, especially in neurological diseases, such as Parkinson’s disease [63, 69].

For Alzheimer’s disease, it has been reported that *CALB1* has protective effects against the pro-apoptotic action of mutant presenilin 1 (PS-1), attenuating the increase in intracellular calcium and aiding in the prevention of impaired mitochondrial function [70]. PS-1 acts by sensitizing cells to apoptosis induced by Aβ peptide, which damages neurons through a mechanism involving disruption of calcium homeostasis and generation of oxidative stress [70]. Thus, regarding *CALB1* down-expression in the SOY1.5 group, we observed that a lower percentage of soybean oil *CALB1* gene is less expressed indicating a negative relationship with this diet and a positive relationship with the neurodegenerative processes.

For the enriched pathway in Fig. 4, IL-5 activates and elevates the expression of *CAST*. The *CAST* binds to and inhibits calpain 1 (mu) in the presence of calcium, which activates and cleaves the apoptosis regulatory protein Bax. The Bax will act by preventing or reducing the frequency, rate, or extent of cell death by apoptotic process [71, 72]. The protein encoded by *CAST* is an endogenous calpain inhibitor and is also related to the proteolysis of amyloid precursor protein. Furthermore, this protein is thought to influence the expression levels of genes that encode structural or regulatory proteins “Neuroscience” and “neurodegenerative diseases” are two related pathways associated with this gene. Related GO annotations of *CAST* include “RNA binding” and “cysteine-type endopeptidase inhibitor activity” [18, 73].

The *CAST* is a cell-permeable peptide that acts as an endogenous inhibitor of calpain in the central nervous system [73, 74]. Calpains are cysteine proteases that are activated by calcium, that is, they are positively regulated by calcium and negatively regulated by *CAST* [75, 76]. These proteases, when in dysregulation of calcium homeostasis, have been implicated in neuronal cell dysfunction and death [76], as well as neurodegenerative diseases [77–79].

Calpains have several important roles such as differentiation, cell attachment motility, signal transduction covering cell signaling pathways, regulation of gene expression and membrane fusion [73, 75]. Furthermore, calpains are reported to play important roles in neuronal functions, implying that the activation of this protease needs to be under a rigid control, which is performed by *CAST*. Thus, the well-known calpain-calpastatin system may be an important target for therapeutic approaches related to neurodegenerative diseases [76].

According to Goll et al. [75], *CAST* is also involved in the regulation of kinases, receptors, and transcription factors. *CAST* expression has been shown to have a neuroprotective effect on cerebral ischemia [80]. In the study of Rao et al. [81], higher expression of *CAST* in JNPL3 (mutant tau P301L) mouse models was used to attenuate calpain expression, which has been linked to the development of tauopathy (neurotoxicity caused by tau protein) and neurodegeneration in Alzheimer’s disease. In an Amyotrophic Lateral Sclerosis mouse model, higher *CAST* expression was associated with neuroprotective effects. According to Rao et al. [82], the *CAST* gene reduces calpain activation, decreases abnormal cytoskeletal protein breakdown, increases survival time, inhibits tau production and *CDK5* activation, and decreases *SOD1*.

The calpain-calpastatin system is also reported in excitotoxicity, a pathological or neurodegenerative process that is initiated by overactivation of neurotransmitters such as glutamate. Excitotoxicity leads to increased cellular calcium levels, which causes activation of various proteases, including calpains [83]. Furthermore, missing *CAST* may impair early stages of neurogenesis [84]. Thus, we observed a higher expression of *CAST* in the SOY1.5 group, that suggests a positive relationship between the gene and the metabolic and oxidative processes found for this group.

The identified network, along with the illustrated genes, corroborate the results found in the pathway maps, indicating that varying the amount of soybean oil in the diet of immunocastrated male pigs influences gene expression in brain tissue. Furthermore, the significance of the detected DEG and their association with intracellular calcium is noteworthy. This processes network (Calcium transport) and the genes enriched in this network corroborate the results found in the pathway maps, indicating that changing the level of soybean oil in pigs’ diet has an effect on gene expression. Therefore, the findings of our study point in a promising direction for furthering our understanding of the pathways and networks associated with calcium-dependent metabolic processes involved in lipid metabolism and oxidative processes. More research is needed to better understand the mechanisms by which dietary factors like FA may influence important physiological processes and gene expression in brain tissue. Understanding the mechanisms involved in calcium homeostasis and energy metabolism in the initiation and progression of neurodegenerative diseases and oxidative/inflammatory processes is extremely important.

## Conclusion

This study showed that different levels of soybean oil in pig diets affect the transcriptomic profile but not the total lipid content or FA profile of brain tissue. The genes, pathways, and networks identified herein play important roles in lipid metabolism, immune response, and calcium transport. Furthermore, because pigs are model animals for human metabolic diseases, the DEG identified, as well as their action in brain tissue, demonstrate the importance of FA in metabolic and oxidative processes. Thus, the current study may help future research in the field of nutrigenomics and help to better understand how the diet, with the inclusion of soybean oil, may influence and modulate biological processes important for brain tissue. Further investigation is required to define what proportion of soybean oil helps in directing and modulating neuroprotection and reducing inflammation in brain tissue.

## Methods

### Ethics Statement

The procedures involving animals were evaluated and approved by the Ethics Committee for the Use of Animals (CEUA, number 2018-28, and protocol 2018.5.1787.11.6) of the Luiz de Queiroz College of Agriculture (ESALQ) – University of São Paulo (USP). All procedures followed the guidelines by the Brazilian Council of Animal Experimentation and the ethical principles in animal research, according to FASS [85], the Guide for the Care and Use of Agricultural Animals in Agricultural Research and Teaching. This study was carried out in compliance with the ARRIVE guidelines.

### Animals, experimental design, and diets

Thirty-six immunocastrated male pigs, the offspring of three sires and thirty-two females of the Large White breed, were used for this study. Pigs were genotyped for the halothane mutation (RYR1 gene) and only homozygous halothane-negative (NN) were used [86]. The pigs had an average body weight of 28.44 ± 2.95 kg and an average age of 71 ± 1.8 days, and were randomly distributed to the treatments during the experimental period of 98 days. Two treatments were used, with six replicate pens per treatment, and three pigs per pen, totalizing 18 pigs per treatment. The pigs *ad libitum* access to feed and water throughout the experimental period, and each pen was equipped with a dry feeder and a nipple drinker. The immunocastration was performed by administering two doses of 2 ml of Vivax® (Pfizer Animal Health, Parkville, Australia) on day 56 (127 days of age) and day 70 (141 days of age), according to the manufacturer’s recommendations.

The experimental diet consisted of a six-phase diet: Grower I - day 0 to 21; Grower II - day 21 to 42; Finisher I - day 42 to 56; Finisher II - day 56 to 63; Finisher III - day 63 to 70; and, Finisher IV - day 70 to 98. Dietary treatments consisted of corn-soybean meal diets either containing 1.5% soybean oil (SOY1.5), a standard diet used in pig production, or containing 3.0% soybean oil (SOY3.0). The diets were formulated to meet or exceed the nutritional requirements according to Rostagno et al. [87], and were provided as a meal form, without antibiotic growth promoters. The diets were formulated to have a similar metabolizable energy content (3.36 Mcal/kg). Details of the diets in this study are adapted and described in Tables S4–S6 [19, 88, 89].

The pigs were slaughtered with a final body weight of 133.9 ± 9.4 kg on day 98 of the experiment. Whole brains of the animals were collected and immediately frozen in liquid nitrogen, transported, and stored in a -80 °C freezer until total RNA extraction. The same portion of the middle region of the frontal lobe was delimited in all brain samples in order to obtain a sample as uniform as possible with the same proportion of white and gray matter and the layers. Complete procedures were described in Almeida et al. [88] and Silva et al. [90].

### Total lipid content and FA profile analyses

For the analysis of total lipid content, 5 g of brain samples were used (in duplicate), which were ground, packed in plastic bags and stored at 4ºC. The ground samples were dried in an oven with air circulation at 105 °C for 12 h. After drying, the samples were packed in filter paper cartridges and placed in a Soxhlet type extraction system. The extraction was conducted with hexane and occurred during six hours, according to the method described by AOAC [91]. The percentage of total lipid in the samples was obtained by the difference between the weight of the flask containing the extracted lipid and the empty flask (previously weighed, the flask was left in an oven at 105 °C for 2 h before each weighing) multiplied by 100.

The FA profile was determined from the total lipid content using 10 g samples. The lipids were cold extracted using the method proposed by Bligh Dyer [92] and the methylation of the samples was performed according to Hartman e Lago [93], with adaptations based on AOCS [94] (method AM 5 − 04). The complete procedures were described by Silva et al. [90] and Almeida et al. [88].

Data were analyzed as a randomized complete block design using the MIXED procedure of SAS (SAS Inst. Inc., Cary, NC), with pen being considered as the experimental unit. The model included the random effects of pen and block and the fixed effects of soybean oil levels. Outliers were removed from the data sets and residuals were tested for a normal distribution using the Shapiro-Wilk test (UNIVARIATE procedure). Means were adjusted by using the LSMEANS statement. Differences were declared significant when *p*-value ≤ 0.05 based on the F-test.

### RNA extraction, library preparation, sequencing and data analysis

For the total RNA extraction from the brain samples, we used the commercial kit for RNA extraction (RNeasy® Mini Kit, Qiagen) and the Trizol reagent (Invitrogen). The inclusion of first step using the Trizol, allowed for better phase separation and thus lipid removal as brain tissue has a large amount of lipids (~ 10%). Quality and concentration of total RNA was obtained by using the NanoDrop 2000 Spectrophotometer (Thermo Fisher Scientific) and Qubit® 2.0 Fluorometer. The RNA integrity was evaluated by using the Agilent 2100 Bioanalyzer (Agilent, Santa Clara-CA, USA). All samples presented an RNA Integrity Number (RIN) greater than or equal to 7.5 (Table S3).

For library preparation, 2 µL of total RNA from each sample was used, according to the protocol described in the TruSeq RNA Sample Preparation kit v2 manual (Illumina, San Diego, CA). The average library size was estimated using the Agilent Bioanalyzer 2100 (Agilent, Santa Clara, CA, USA) and the libraries were quantified using quantitative PCR with the quantification kit, from the KAPA library (KAPA Biosystems, Foster City, CA, USA). TruSeq PE Cluster kit v3-cBot-HS (Illumina, San Diego, CA, USA) was used for the sequencing. The samples were pooled and sequenced by using the HiSeq 2500 equipment (Illumina, San Diego, CA, USA) with a TruSeq SBS Kit v3-HS (200 cycles), according to the manufacturer’s instructions. All sequencing steps were performed at the ESALQ-USP Animal Genomics Center, located in the Animal Biotechnology Laboratory of ESALQ-USP, Piracicaba, São Paulo, Brazil.

For the steps of quality control, low complexity reads and adapters were removed using Trim Galore software (v.0.6.5). The minimum length of reads after removal was 70 bases, with Phred Score lower than 33. Quality control was done by using FastQC software (v.0.11.8) [http://www.bioinformatics.bbsrc.ac.uk/projects/fastqc/]. The reference genome used was the *Sus Scrofa 11.1*, available from Ensembl [http://www.ensembl.org/Sus_scrofa/Info/Index]. Alignment, mapping, and abundance (read counts) of mRNAs for all known-genes was performed using STAR software (v.2.7.6a) [95], and the gene expression levels were normalized using the counts scaled by total number of reads or counts per million (CPM).

### Identification of differentially expressed genes, and functional enrichment analysis

The differentially expressed genes (DEG) between the SOY1.5 and SOY3.0 groups were identified by using the DESeq2 statistical package (R/Bioconductor) [http://www.bioconductor.org/packages/release/bioc/html/DESeq2.html], using a multi-factor design [96]. Before statistical analysis, filtering criteria were used: (i) removal of genes with zero counts for all samples, that is, unexpressed genes, (ii) removal of genes with less than one read per sample on average were removed (very lowly expressed); (iii) removal of genes that were not present in at least 50% of the samples were removed (rarely expressed). The model used, included treatments as the variable of interest and father as a fixed effect. Correction for multiple testing was performed, according to the False Discovery Rate (FDR) method [97], and the threshold value used for significance was FDR < 0.05.

The enrichment analysis was performed using the MetaCore software (Clarivate Analytics, London, UK, v.21.4, build 70,700) [https://clarivate.com/products/metacore/]. The pathway maps were identified from the list of known-genes DEG obtained from SOY1.5 vs. SOY3.0 (FDR < 0.05) comparison. For annotation and functional enrichment, the *Homo sapiens* genome was used as background reference. Functional enrichment analysis to obtain comparative pathways and networks was performed, using the standard parameter. The filters for the metabolic maps of interest were used: energy metabolism, lipid metabolism, steroid metabolism, regulation of cellular processes (immune response, neurophysiological process, and oxidative stress), regulation of metabolism, mental disorders, nutritional and metabolic diseases, nervous system diseases, and tox processes. To understand the behavior of genes and their interactions, networks were created.

## Electronic supplementary material

Below is the link to the electronic supplementary material.

**Additional file 1.** **Table S1.** Total reads in brain samples of pigs fed diets containing different levels of soybean oil.

**Additional file 2**. **Table S2.** Differentially expressed genes in brain tissue of pigs fed diets containing different levels of soybean oil.

**Additional file 3.** **Table S3.** Quality, concentrattion and RNA integrity number of brain samples of pigs fed diets containing different levels of soybean oil.

**Additional file 4. Table S4.** Composition of the experimental diets (as-fed basis). **Table S5.** Analyzed fatty acid profile of grower diets (as-fed basis).** Table S6.** Analyzed fatty acid profile of finisher diets (as-fed basis).

## Data Availability

The dataset supporting the conclusions of this article is available in the European Nucleotide Archive (ENA) repository (EMBL-EBI), under accession PRJEB52665 [http://www.ebi.ac.uk/ena/data/view/PRJEB52665]. The original contributions presented in the study are included in the article and supplementary material, further inquiries can be directed to the corresponding author.
