# Supplementary material for: Brain fatty acid and transcriptome profiles of pig fed diets with different levels of soybean oil
Source: BMC Genomics. 2023 Feb 28;24:91. doi: 10.1186/s12864-023-09188-6 (PMC9976441; doi:10.1186/s12864-023-09188-6)
Supplement: Supplementary file 4 — Additional file 4. Table S4. Composition of the experimental diets (as-fed basis). Table S5. Analyzed fatty acid profile of grower diets (as-fed basis). Table S6. Analyzed fatty acid profile of finisher diets (as-fed basis). [file 12864_2023_9188_MOESM4_ESM.docx]

Table S4. Composition of the experimental diets (as-fed basis)

|  | Grower I | |  | Grower II | |  | Finisher I | |  | Finisher II | |  | Finisher III | |  | Finisher IV | |
| --- | --- | --- | --- | --- | --- | --- | --- | --- | --- | --- | --- | --- | --- | --- | --- | --- | --- |
|  | (day 0 to 21) | |  | (day 21 to 42) | |  | (day 42 to 56) | |  | (day 56 to 63) | |  | (day 63 to 70) | |  | (day 70 to 98) | |
| Item | SOY1.5 | SOY3.0 |  | SOY1.5 | SOY3.0 |  | SOY1.5 | SOY3.0 |  | SOY1.5 | SOY3.0 |  | SOY1.5 | SOY3.0 |  | SOY1.5 | SOY3.0 |
| Ingredient, % |  |  |  |  |  |  |  |  |  |  |  |  |  |  |  |  |  |
| Corn, 7.5% CP^1^ | 63.47 | 61.88 |  | 66.40 | 64.71 |  | 69.13 | 67.54 |  | 69.63 | 68.04 |  | 69.59 | 68.00 |  | 70.09 | 68.50 |
| Soybean meal, 46% CP | 28.33 | 28.42 |  | 26.10 | 26.29 |  | 23.37 | 23.46 |  | 23.37 | 23.46 |  | 22.93 | 23.02 |  | 22.93 | 23.02 |
| Meat and bone meal, 44% CP | 3.00 | 3.00 |  | 3.00 | 3.00 |  | 3.00 | 3.00 |  | 3.00 | 3.00 |  | 3.00 | 3.00 |  | 3.00 | 3.00 |
| Fat source^2^ | 1.50 | 3.00 |  | 1.50 | 3.00 |  | 1.50 | 3.00 |  | 1.50 | 3.00 |  | 1.50 | 3.00 |  | 1.50 | 3.00 |
| Dicalcium phosphate | 0.55 | 0.56 |  | 0.56 | 0.57 |  | 0.26 | 0.27 |  | 0.26 | 0.27 |  | 0.27 | 0.27 |  | 0.26 | 0.27 |
| Limestone | 0.43 | 0.42 |  | 0.38 | 0.38 |  | 0.84 | 0.84 |  | 0.76 | 0.75 |  | 0.84 | 0.84 |  | 0.69 | 0.69 |
| Salt | 0.50 | 0.50 |  | 0.50 | 0.50 |  | 0.50 | 0.50 |  | 0.50 | 0.50 |  | 0.50 | 0.50 |  | 0.50 | 0.50 |
| Vitamin-mineral premix^3^ | 1.61 | 1.61 |  | 1.08 | 1.08 |  | 1.01 | 1.01 |  | 0.60 | 0.60 |  | 1.02 | 1.01 |  | 0.66 | 0.65 |
| L-Lysine.HCl | 0.35 | 0.35 |  | 0.29 | 0.29 |  | 0.25 | 0.25 |  | 0.25 | 0.25 |  | 0.20 | 0.20 |  | 0.20 | 0.20 |
| DL-Methionine | 0.11 | 0.11 |  | 0.07 | 0.08 |  | 0.04 | 0.04 |  | 0.04 | 0.04 |  | 0.02 | 0.02 |  | 0.03 | 0.03 |
| L-Threonine | 0.14 | 0.15 |  | 0.11 | 0.11 |  | 0.09 | 0.09 |  | 0.09 | 0.09 |  | 0.06 | 0.06 |  | 0.06 | 0.07 |
| L-Tryptophan | 0.01 | 0.01 |  | - | - |  | - | - |  | - | - |  | - | - |  | - | - |
| Ractopamine.HCl, 2% | - | - |  | - | - |  | - | - |  | - | - |  | 0.08 | 0.08 |  | 0.08 | 0.08 |
| Calculated composition^4^ |  |  |  |  |  |  |  |  |  |  |  |  |  |  |  |  |  |
| Metabolizable energy, Mcal/kg | 3.28 | 3.36 |  | 3.29 | 3.36 |  | 3.28 | 3.36 |  | 3.29 | 3.36 |  | 3.28 | 3.35 |  | 3.29 | 3.36 |
| SID^5^ Lysine, % | 1.15 | 1.15 |  | 1.05 | 1.05 |  | 0.95 | 0.95 |  | 0.95 | 0.95 |  | 0.90 | 0.90 |  | 0.90 | 0.90 |
| SID Methionine + Cysteine, % | 0.62 | 0.62 |  | 0.57 | 0.57 |  | 0.51 | 0.51 |  | 0.51 | 0.51 |  | 0.49 | 0.49 |  | 0.49 | 0.49 |
| SID Threonine, % | 0.75 | 0.75 |  | 0.68 | 0.68 |  | 0.63 | 0.63 |  | 0.63 | 0.63 |  | 0.58 | 0.59 |  | 0.59 | 0.59 |
| SID Tryptophan, % | 0.22 | 0.22 |  | 0.20 | 0.20 |  | 0.18 | 0.18 |  | 0.18 | 0.18 |  | 0.18 | 0.18 |  | 0.18 | 0.18 |
| Calcium, % | 0.84 | 0.84 |  | 0.81 | 0.81 |  | 0.80 | 0.80 |  | 0.77 | 0.77 |  | 0.80 | 0.80 |  | 0.75 | 0.75 |
| Available Phosphorous, % | 0.42 | 0.42 |  | 0.42 | 0.42 |  | 0.34 | 0.34 |  | 0.34 | 0.34 |  | 0.34 | 0.34 |  | 0.34 | 0.34 |
| Analyzed composition, % |  |  |  |  |  |  |  |  |  |  |  |  |  |  |  |  |  |
| CP | 19.71 | 19.64 |  | 18.75 | 18.71 |  | 17.66 | 17.58 |  | 17.66 | 17.59 |  | 17.41 | 17.33 |  | 17.42 | 17.35 |
| Ether extract | 4.49 | 5.49 |  | 4.03 | 5.53 |  | 3.63 | 6.57 |  | 3.63 | 5.59 |  | 3.16 | 5.61 |  | 3.16 | 5.61 |

^1^CP = crude protein. ^2^Corn-soybean meal diet containing 1.5% soybean oil (SOY1.5) or corn-soybean meal diet containing 3% soybean oil (SOY3.0); ^3^Provided per kilogram of diet: 6.500 UI vitamin A; 1.800 UI vitamin D_3_; 30 UI vitamin E; 2 mg vitamin K_3_; 1.2 mg vitamin B_1_; 3.4 mg vitamin B_2_; 2.0 mg vitamin B_6_; 125 mg Cu; 80 mg Fe; 40 mg Mn; 0.35 mg Se; 1.25 mg Zn. ^4^Calculated according to Rostagno et al. [86]. ^5^SID = standardized ileal digestible. Adapted from Almeida et al. [83] and Fanalli et al. [85].

Table S5. Analyzed fatty acid profile of grower diets (as-fed basis)

|  | Grower I (day 0 to 21) | | | | Grower II (day 21 to 42) | | |  |
| --- | --- | --- | --- | --- | --- | --- | --- | --- |
| Fatty acid, % | SOY1.5^1^ | | SOY3.0^2^ |  | | SOY1.5 | SOY3.0 | |
| Saturated fatty acid (SFA) |  |  | |  | |  |  | |
| Myristic acid (C14:0) | ND^3^ | 1.85 | |  | | 0.24 | 0.31 | |
| Palmitic acid (C16:0) | 13.97 | 12.76 | |  | | 13.49 | 14.37 | |
| Margaric acid (C17:0) | ND | ND | |  | | 0.15 | ND | |
| Stearic acid (C18:0) | 4.21 | 2.44 | |  | | 4.01 | 4.50 | |
| Arachidic acid (C20:0) | 0.46 | ND | |  | | 0.48 | 0.45 | |
| Behenic acid (C22:0) | 0.33 | ND | |  | | 0.22 | 0.24 | |
| Monounsaturated fatty acid (MUFA) |  |  | |  | |  |  | |
| Palmitoleic acid (C16:1) | 0.30 | ND | |  | | 0.19 | 0.26 | |
| Oleic acid (C18:1 n-9) | 28.97 | 23.27 | |  | | 33.21 | 31.92 | |
| Eicosenoic acid (C20:1 n-9) | 0.27 | ND | |  | | 0.28 | 0.21 | |
| Polyunsaturated fatty acid (PUFA) |  |  | |  | |  |  | |
| Linoleic acid (C18:2 n-6) | 47.62 | 55.65 | |  | | 45.33 | 44.80 | |
| Alpha-linolenic acid (C18:3 n-3) | 3.57 | 5.85 | |  | | 2.41 | 2.95 | |
| Eicosapentaenoic acid (C20:5 n-3) | ND | ND | |  | | ND | ND | |
| Docosahexaenoic acid (C22:6 n-3) | ND | ND | |  | | ND | ND | |
| Total SFA | 18.97 | 17.05 | |  | | 18.59 | 19.87 | |
| Total MUFA | 29.54 | 23.27 | |  | | 33.68 | 32.39 | |
| Total PUFA | 51.19 | 61.5 | |  | | 47.74 | 47.75 | |
| PUFA:SFA ratio^4^ | 2.70 | 3.61 | |  | | 2.57 | 2.40 | |

^1^SOY1.5 = corn-soybean meal diet containing 1.5% soybean oil; ^2^SOY3.0 = corn-soybean meal diet containing 3% soybean oil; ^3^ND = not detectable. ^4^PUFA:SFA ratio = total PUFA/total SFA. Adapted from Almeida et al. [83] and Fanalli et al. [85].

Table S6. Analyzed fatty acid profile of finisher diets (as-fed basis)

|  | Finisher I (day 42 to 56) | |  | Finisher II (day 56 to 63) | |  | Finisher III (day 63 to 70) | |  | Finisher IV (day 70 to 98) | |
| --- | --- | --- | --- | --- | --- | --- | --- | --- | --- | --- | --- |
| Fatty acid, % | SOY1.5^1^ | SOY3.0^2^ |  | SOY1.5 | SOY3.0 |  | SOY1.5 | SOY3.0 |  | SOY1.5 | SOY3.0 |
| Saturated fatty acid (SFA) |  |  |  |  |  |  |  |  |  |  |  |
| Myristic acid (C14:0) | 0.29 | 1.87 |  | ND | ND |  | 0.43 | 0.28 |  | ND | ND |
| Palmitic acid (C16:0) | 13.62 | 19.75 |  | 11.88 | 12.59 |  | 13.61 | 13.82 |  | 12.90 | 14.45 |
| Margaric acid (C17:0) | ND^2^ | 0.34 |  | ND | ND |  | ND | 0.16 |  | ND | ND |
| Stearic acid (C18:0) | 4.29 | 4.96 |  | 3.13 | 2.83 |  | 4.98 | 4.28 |  | 3.81 | 4.53 |
| Arachidic acid (C20:0) | 0.43 | 0.43 |  | 0.42 | 0.41 |  | 0.40 | 0.43 |  | ND | 0.43 |
| Behenic acid (C22:0) | ND | ND |  | 0.32 | 0.21 |  | ND | 0.19 |  | ND | ND |
| Monounsaturated fatty acid (MUFA) |  |  |  |  |  |  |  |  |  |  |  |
| Palmitoleic acid (C16:1) | 0.21 | 3.49 |  | 0.11 | 0.11 |  | 0.54 | 0.32 |  | ND | 0.22 |
| Oleic acid (C18:1 n-9) | 35.64 | 36.44 |  | 30.79 | 34.84 |  | 33.32 | 34.95 |  | 30.18 | 35.58 |
| Eicosenoic acid (C20:1 n-9) | ND | ND |  | 0.20 | 0.23 |  | 0.32 | 0.26 |  | 0.42 | ND |
| Polyunsaturated fatty acid (PUFA) |  |  |  |  |  |  |  |  |  |  |  |
| Linoleic acid (C18:2 n-6) | 42.82 | 27.52 |  | 48.90 | 46.03 |  | 42.63 | 42.85 |  | 48.53 | 42.56 |
| Alpha-linolenic acid (C18:3 n-3) | 2.70 | 1.65 |  | 4.26 | 2.76 |  | 3.78 | 2.45 |  | 4.16 | 2.24 |
| Eicosapentaenoic acid (C20:5 n-3) | ND | ND |  | ND | ND |  | ND | ND |  | ND | ND |
| Docosahexaenoic acid (C22:6 n-3) | ND | ND |  | ND | ND |  | ND | ND |  | ND | ND |
| Total SFA | 18.63 | 27.35 |  | 15.75 | 16.04 |  | 19.42 | 19.16 |  | 16.71 | 19.41 |
| Total MUFA | 35.85 | 39.93 |  | 31.1 | 35.18 |  | 34.18 | 35.53 |  | 30.6 | 35.8 |
| Total PUFA | 45.52 | 29.17 |  | 53.16 | 48.79 |  | 46.41 | 45.3 |  | 52.69 | 44.8 |
| PUFA:SFA ratio^4^ | 2.44 | 1.07 |  | 3.38 | 3.04 |  | 2.39 | 2.36 |  | 3.15 | 2.31 |

^1^SOY1.5 = corn-soybean meal diet containing 1.5% soybean oil; ^2^SOY3.0 = corn-soybean meal diet containing 3% soybean oil; ^3^ND = not detectable. ^4^PUFA:SFA ratio = total PUFA/total SFA. Adapted from Almeida et al. [83] and Fanalli et al. [85].
